# Method to determine the decrepitation index of South African manganese ores when heating in a rotary kiln

**DOI:** 10.1016/j.mex.2022.101720

**Published:** 2022-05-04

**Authors:** M.S. Moholwa, J.D. Steenkamp, H.L. Rutto

**Affiliations:** 1 Mintek, 200 Malibongwe Drive, Randburg, 2125, South Africa; 2 Vaal University of Technology, Andries Potgieter Blvd, Vanderbijlpark, 1900, South Africa; 3 University of the Witwatersrand, 1 Jan Smuts Avenue, Johannesburg, 2000, South Africa

**Keywords:** Decrepitation Index, Manganese ore, Temperature, Rotational speed, Particle size range

## Abstract

Manganese ores are the major raw materials utilized in the production of manganese ferroalloys. Decrepitation, which is described as the breakage or disintegration of the ore particles upon heating in a rotary kiln, is an important quality parameter of these ores. The Decrepitation Index (DI), the parameter used to describe the extent of decrepitation, is described as the ratio of the mass of particles <6 mm after pre-heating to the total mass of the sample. After a rigorous review of literature, it was found that the procedure to determine DI of manganese ores in rotary kiln does not exist. This necessitated the development of a method to determine the DI of manganese ores when pre-heated in a rotary kiln. The method was developed through the following steps

• The method from the ISO standard 8371 to determine the DI of iron ores in a muffle furnace was studied and the method developed was based on the method.
• A literature review on the decrepitation of ores and the factors that cause fines generation during a tumbling test.

The method developed can be used to investigate the effect of temperature, rotational speed and size range on the DI of South African manganese ores.

## Graphical abstract

**Figure:**
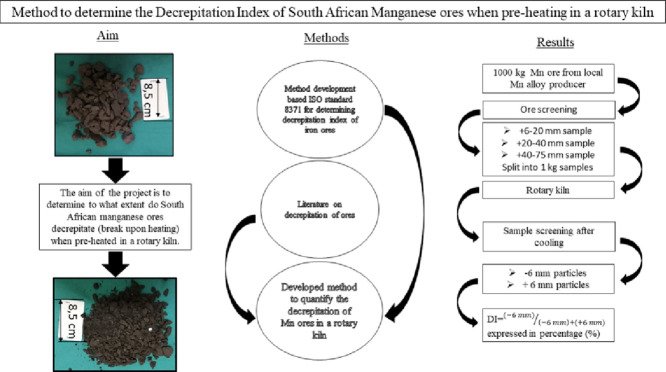

Specifications table

**Table:** 

Subject Area:	
More specific subject area:	*Production of High Carbon Ferromanganese Alloys (HCFeMn)*
Method name:	*Method to determine the decrepitation index of South African Manganese ores when pre-heating in a rotary kiln*
Name and reference of original method:	*ISO 8371:2004 – Iron ores – Determination of decrepitation index*
Resource availability:	N/A

## Method details

The method was adopted from ISO 8371 (2004) [1] which is a method used to quantify the decrepitation index of iron ores in a muffle furnace at 700°C, as well as other source of literature on factors causing fines generation during a tumbling test (Kingman et al., 2008 [2]). The method was then adapted to suit the objectives of determining the effect of temperature, rotational speed, and particle size range on the DI of South African manganese ores when pre-heated in a rotary kiln. The method involved utilisation of an in-house built rotary kiln externally heated with electrical elements. The kiln power was switched on and a temperature set point of either 600, 800 or 1000°C was programmed. The rotational speed was set to either 3, 6 or 12 rpm. The kiln was allowed to slowly warm up and reach the intended temperature for the specific test, the heating rate for all the tests was 2.5°C/minute under atmospheric pressure and in air The kiln heating was 240 minutes, 320 minutes and 400 minutes to heat up to 600, 800 and 1 000°C, respectively. As soon as the kiln has reached the intended temperature, 1 kg of manganese ore of either the size range of +6-20, +20-40 or +40-75 mm was fed into the kiln and left to heat up and tumble for 30 minutes. After the 30 minutes, the kiln power and rotation were switched off and the sample allowed to cool overnight in the kiln. The following day the sample was removed by tilting the rotating tube and collecting all the sample. The sample was then weighed and screened with a 6 mm sieve manufactured by Kingtest with a model number BS410:19986 and sieve shaker manufactured by Laarmann with a model number LMSM-75/200 to get the mass of particles that were <6 mm. The sieve shaker operated for a duration of 2 minutes for each sample. The weight of <6 mm particles was divided by the total weight of the sample (which includes >6 mm particles) to get the DI. The DI was determined using the equation displayed in Eq. [1].

(1) DI(<6mm)=(M1/M2)X100%

Where:

• DI = Decrepitation index (%)
• M1 = Mass of particles <6 mm after heating (g)
• M2 = Total mass of the sample after heating (g)

The moisture content of the three ores was determined through drying of the material overnight. 1 kg of each ore type was weighed and placed in the drying oven at 105°C. The samples were left in the digital drying oven manufactured by WIRSAM SCIENTIFIC with a model number 276 overnight and the following day the sample was removed and left to cool in air. After cooling the samples were weighed. The moisture content was calculated as per Equation 1.

Equation 1

$$M=\;\frac{a}{b}x100\%$$

Where:

• M = Moisture content (%)
• a = Mass lost during drying (g)
• b = Mass before drying (g)

**Fig. 1:**
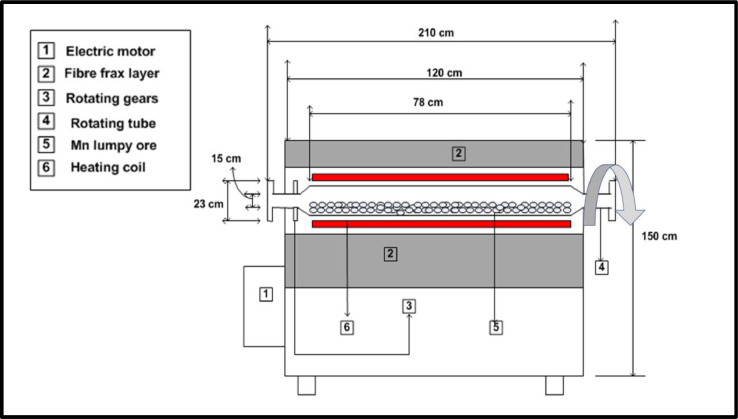
Experimental set-up

The experimental set-up and the experimental plan are displayed Fig. 1 and Table 1, respectively. The custom-made rotary kiln, with a 316 stainless steel rotating tube, is equipped with a REX-P200 temperature controller. The kiln can provide a maximum heating rate of 10 °C/minute and can reach a maximum temperature of 1 200 °C. The kiln is also equipped with a Direct Current (DC) motor and a variable speed drive which can give a maximum output of 12 rpm. The rotating tube and DC motor are connected by means of gears and chains. The cross-sectional view of the rotary kiln coupled with the dimensions are displayed in Fig. 1.

**Table 1:** Experimental plan for each ore type

Test no	Particle size range (mm)	Rotational speed (rpm)	Temperature (°C)
1	+6-20	6	800
2	+20-40	6	800
3	+40-75	6	800
4	+6-20	3	800
5	+6-20	6	800
6	+6-20	12	800
7	+6-20	6	600
8	+6-20	6	800
9	+6-20	6	1 000

Supplementary material *and/or* Additional information

At the beginning of the project, the effect of heating rate on the decrepitation index of manganese ores was included in the objectives. The procedure involved heating up the ore at different heating rates while keeping every other parameter constant. 1 kg of the sample was fed into the kiln at a temperature set-point of 800 °C and heating rates of 2.5, 5 or 7.5 °C/minute. The sample was heated to the temperature of 800 °C and held for 30 minutes. After 30 minutes, the sample was allowed to cool inside the kiln overnight. The sample was then removed from the kiln and screened for particles <6 mm to obtain the DI. The flaw with the procedure was that changing the heating rate changes the tumbling duration. The test that utilizes a heating rate of 2.5 °C/minute will tumble for a longer duration when compared to the test that utilized a heating rate of 7.5 °C/minute. Therefore, the changes that were observed in these tests were not solely due to changing the heating rate but also due to tumbling time. For this reason, there was a need to modify the procedure. It was proposed to heat up the sample with the kiln stationary and only switch on the rotation for 30 minutes when the sample is at temperature. This idea was however hindered by a safety engineering design installed on the kiln to stop the kiln from heating up when the kiln rotation is off, and therefore the method described in the “method details” heading was implemented.

## Appendix Supplementary materials

Image, video 1
